# Risk factors for vancomycin treatment failure in heterogeneous vancomycin-intermediate *Staphylococcus aureus* bacteremia

**DOI:** 10.1128/spectrum.00333-24

**Published:** 2024-06-25

**Authors:** Ji Hyun Yun, Euijin Chang, Seongman Bae, Jiwon Jung, Min Jae Kim, Yong Pil Chong, Sung-Han Kim, Sang-Ho Choi, Sang-Oh Lee, Yang Soo Kim

**Affiliations:** 1 Division of Infectious Diseases, Department of Internal Medicine, Konkuk University Medical Center, Konkuk University School of Medicine, Seoul, South Korea; 2 Division of Infectious Diseases, Asan Medical Center, University of Ulsan College of Medicine, Seoul, South Korea; 3 Center for Antimicrobial Resistance and Microbial Genetics, University of Ulsan College of Medicine, Seoul, South Korea; Icahn School of Medicine at Mount Sinai, New York, New York, USA

**Keywords:** hVISA, vancomycin, treatment failure, risk factors, age, severity, minimal inhibitory concentration

## Abstract

The incidence of heterogeneous vancomycin-intermediate *Staphylococcus aureus* (hVISA) infection is increasing and is associated with vancomycin treatment failures. However, studies investigating the risk factors for treatment failure in hVISA infection are limited. Patients with hVISA bacteremia treated with vancomycin over 7 days between August 2008 and June 2020 were enrolled in this study. Clinical and microbiological characteristics were compared between vancomycin treatment failure and success groups to identify the risk factors for vancomycin treatment failure. Among the 180 patients with hVISA bacteremia, 102 patients treated with vancomycin over 7 days were included. Vancomycin treatment failed in 80 (78%) patients. Patients in the vancomycin treatment failure group were older (*P* < 0.001) and more frequently had solid cancer (*P* = 0.04) than those in the vancomycin treatment success group. Solid organ transplantation (SOT) was more frequent (*P* < 0.001) in the vancomycin treatment success group. The Charlson comorbidity index (*P* = 0.01) and Acute Physiology and Chronic Health Evaluation II scores (*P* < 0.001) were higher in the vancomycin treatment failure group. In multivariate analysis, independent risk factors for vancomycin treatment failure were old age and severity of bacteremia. SOT and vancomycin minimal inhibitory concentration (MIC) ≤ 1.0 mg/L using the broth microdilution (BMD) method were associated with successful vancomycin treatment. Old age and infection severity were independent risk factors for vancomycin treatment failure. Vancomycin MIC using the BMD method is an important risk factor for vancomycin treatment failure, and its use should be considered in hVISA bacteremia.

**IMPORTANCE:**

In this study, we assessed the clinical and microbiological characteristics of heterogeneous vancomycin-intermediated *Staphylococcus aureus* (hVISA) bacteremia and identified risk factors for vancomycin treatment failure. We found that advanced age and severity of infection were independent risk factors for vancomycin treatment failure. On the other hand, solid organ transplantation and a low vancomycin minimal inhibitory concentration were associated with successful vancomycin treatment. This study highlights the importance of vancomycin minimal inhibitory concentration in hVISA bacteremia.

## INTRODUCTION

Methicillin-resistant *Staphylococcus aureus* (MRSA) infections are significant in both nosocomial and community-acquired infections (1). Because vancomycin is the first-line treatment of MRSA infection, the use of vancomycin was increased. This increase in vancomycin use has led to a rise in vancomycin-resistant strains, including heterogeneous vancomycin-intermediate *S. aureus* (hVISA) and vancomycin-intermediated *S. aureus* (VISA) (2–5).

First identified in 1997, MRSA strains such as hVISA and VISA exhibit reduced vancomycin susceptibility (6, 7). The prevalence of the hVISA phenotype among MRSA isolates varies widely, reported between 0% and 73% (3) globally and 2.7% and 37% in Korea (8, 9), largely owing to differing hVISA definitions. hVISA is significantly associated with vancomycin treatment failure in MRSA bacteremia, but not necessarily with increased mortality (3). However, a recent study did link hVISA with higher mortality in MRSA pneumonia (10), though findings on poor outcomes in MRSA bacteremia are inconsistent (8, 11–13).

Thus, elucidating the risk factors for poor outcomes in hVISA infection is important, but few studies have been performed on this line. Chong et al. (14) reported the severity of underlying diseases and the severity of infection as risk factors for mortality in patients with hVISA bacteremia. A study reported comorbidities, disease severity, and hVISA phenotype as risk factors for vancomycin treatment failure in bacteremia due to MRSA with reduced vancomycin susceptibility (15); however, because only 37% of hVISA isolates were included among the MRSA strains with reduced vancomycin susceptibility, it is difficult to extrapolate the results of this study to hVISA infection.

In this study, we aimed to evaluate the clinical and microbiological characteristics of patients with hVISA bacteremia treated with vancomycin, compare treatment outcomes, and identify risk factors for vancomycin treatment failure in hVISA bacteremia.

## RESULTS

### Study population

During the study, 180 of 924 (19%) patients with MRSA bacteremia were identified to have the hVISA phenotype. Among them, 102 patients were treated with vancomycin for over 7 days, as definitive therapy, and were enrolled in the study. We excluded 78 patients treated with vancomycin for less than 7 days (49 patients), teicoplanin (18 patients), linezolid (4 patients), tigecycline (3 patients), clindamycin (1 patient), levofloxacin (1 patient), and non-susceptible antibiotics (1 patient). The details of the 49 patients who were treated with vancomycin for less than 7 days were as follows: changed to other antibiotics (38 patients), transferred to other hospitals (4 patients), discharged (3 patients), death (2 patients), and unknown causes (2 patients). Vancomycin failure was observed in 80 (78%) patients. Specifically, 69 (68%) patients experienced all-cause mortality, 35 (34%) patients had persistent bacteremia (over 3 days despite vancomycin treatment), and 4 (4%) patients had recurrent bacteremia. The remaining 22 patients, not meeting the criteria of vancomycin failure, were thus classified into the vancomycin success group.

### Characteristics of the hVISA cohort

The median age of the entire cohort was 65 years [interquartile range (IQR), 55–72], and 82 of 102 patients (80%) were male. Nosocomial infections dominated at 82%, with community-acquired infections at 2%. The prevalent underlying diseases were hypertension (46%), diabetes (36%), and solid cancers (27%). Sixty-four patients (63%) had central venous catheters (CVC) at the time of bacteremia. Foreign materials other than CVC were inserted in 69 (68%) patients. Catheters other than vascular catheters, such as biliary catheters, urinary catheters, and other drainage catheters, were inserted in 53 (52%) patients, and foreign materials other than catheters, such as prosthetic valves, prosthetic joints, and vascular stents, were present in 20 (20%) patients. The median Acute Physiology and Chronic Health Evaluation II (APACHE II) scores at bacteremia were 16 (IQR, 12–21), Pitt bacteremia score was 1 (IQR, 0–3), and septic shock was observed in nine (9%) patients. Catheter-related bloodstream infection (CRBSI) (48%), primary bacteremia (15%), and pneumonia (12%) were the most common sites of infection. Bone and joint infection and infective endocarditis were observed in 10 (10%) and 4 (4%) patients, respectively (Table 1).

**TABLE 1:** Clinical characteristics of the patients with heterogeneous vancomycin-intermediate *Staphylococcus aureus* bacteremia who were treated with vancomycin^*d*^

Characteristics	Total, *n* = 102	Vancomycin success, *n* = 22	Vancomycin failure, *n* = 80	*P-*value
Age, median (IQR) (years)	65 (55–72)	51 (48–59)	68 (59–73)	<0.001
Male	82 (80)	19 (86)	63 (79)	0.55
Place of acquisition				0.36
Community-acquired	2 (2)	1 (5)	1 (1)	
Healthcare-associated	16 (16)	4 (18)	12 (15)	
Nosocomial	84 (82)	17 (77)	67 (84)	
Underlying disease				
Hypertension	47 (46)	5 (23)	42 (53)	0.01
Solid cancer	38 (37)	4 (18)	34 (43)	0.04
Diabetes mellitus	37 (36)	6 (27)	31 (39)	0.32
Ischemic heart disease	16 (16)	0	16 (20)	0.02
Cerebrovascular accident	17 (17)	1 (5)	16 (20)	0.11
End-stage renal disease	13 (13)	1 (5)	12 (15)	0.29
Liver cirrhosis	10 (10)	5 (23)	5 (6)	0.04
Solid organ transplantation	12 (12)	8 (36)	4 (5)	<0.001
Hematologic malignancy	3 (3)	0	3 (4)	>0.99
HSCT	2 (2)	0	2 (3)	>0.99
Underlying condition				
Recent surgery	42 (41)	15 (68)	27 (34)	0.004
Immunosuppressed status	27 (27)	9 (41)	18 (23)	0.08
ICU admission	28 (28)	6 (27)	22 (28)	0.98
Chemotherapy	5 (5)	1 (5)	4 (5)	>0.99
Neutropenia	2 (2)	0	2 (3)	>0.99
Presence of CVC	64 (63)	14 (64)	50 (63)	0.92
Foreign materials other than CVC				
Catheters^*a*^	53 (52)	11 (50)	42 (53)	0.84
Non-catheters^*b*^	20 (20)	6 (27)	14 (18)	0.37
McCabe Jackson				0.48
Nonfatal	64 (63)	16 (73)	48 (60)	
Ultimately fatal	37 (36)	6 (27)	31 (39)	
Rapid fatal	1 (1)	0	1 (1)	
Charlson comorbidity, median (IQR)	3 (2–5)	2 (1–3)	3 (2–5)	0.01
MRSA colonization	54 (53)	8 (36)	46 (58)	0.08
APACHE II, at bacteremia, median (IQR)	16 (12–21)	11 (9–15)	17 (13–22)	<0.001
Pitt bacteremia score, median (IQR)	1 (0–3)	0 (0–1)	1 (0–3)	0.07
Septic shock	9 (9)	1 (5)	8 (10)	0.68
Site of infection				
CRBSI	49 (48)	10 (46)	39 (49)	0.78
Pneumonia	12 (12)	0	12 (15)	0.07
Surgical site infection	12 (12)	4 (18)	8 (10)	0.28
Skin and soft tissue infection	10 (10)	1 (5)	9 (11)	0.69
Bone and joint infection	10 (10)	1 (5)	9 (11)	0.69
Infective endocarditis	4 (4)	0	4 (5)	0.58
Others^*c*^	7 (7)	2 (9)	5 (6)	0.64
Primary bacteremia	15 (15)	4 (18)	11 (14)	0.73
Eradicable focus	72 (71)	16 (73)	56 (70)	0.80
Removal of focus	66/72	16/16	50/56	0.33
Interval from bacteremia to removal, median (IQR), days	2 (0–4)	3 (1–5)	2 (0–3)	0.07

^*a*^ This included catheters other than vascular catheters, such as biliary catheters, urinary catheters, and other drainage catheters.

^*b*^ This included foreign materials other than catheters, such as prosthetic valves, vascular stents, and prosthetic joints.

^*c*^ Other focuses included endophthalmitis, urinary tract infection, central nervous system infection, and others.

^*d*^ HSCT, hematopoietic stem cell transplantation; ICU, intensive care unit.

Vancomycin minimal inhibitory concentration (MIC) was categorized as ≤1.0, 1.5, and ≥2.0 mg/L. Using E-test, vancomycin MIC was ≤1.0 mg/L in 15 (15%) isolates, 1.5 mg/L in 46 (45%) isolates, and ≥2.0 mg/L in 41 (40%) isolates. Detailed vancomycin MIC of 41 isolates with vancomycin ≥2.0 mg/L was 2.0 mg/L in 31 isolates, 3.0 mg/L in 9 isolates, and 4.0 mg/L in 1 isolate. Using the broth microdilution (BMD) method, vancomycin MIC was ≤1.0 mg/L in 57 (56%) isolates, 1.5 mg/L in 34 (33%) isolates, and ≥2.0 mg/L in 11 (11%) isolates. Detailed vancomycin MIC of 11 isolates with vancomycin ≥2.0 mg/L were 2.0 mg/L in 10 isolates and 4.0 mg/L in 1 isolate. Vancomycin MIC of isolates with vancomycin ≤ 1.0 mg/L was all 1.0 mg/L in the E-test and BMD method. In the automated test, vancomycin MIC was ≤1.0 mg/L in 52 (51%) isolates and ≥2.0 mg/L in 50 isolates (49%). Among the isolates with vancomycin MIC ≤ 1.0 mg/L, isolates with vancomycin MIC 0.5 and 1.0 mg/L were 6 (6%) and 46 (45%), respectively. The vancomycin MIC of isolates with vancomycin MIC ≥2.0 mg/L was all 2.0 mg/L. Isolates were found to be resistant to various antimicrobial agents, including clindamycin, ciprofloxacin, erythromycin, and tetracycline. However, unlike the rate of resistance to other antimicrobial agents, the rate of resistance to rifampicin (16%) and trimethoprim/sulfamethoxazole (11%) was relatively low. Sequence type (ST) 5 was the major strain (83%), *agr* genotype II was the most frequent (85%), and *agr* dysfunction was detected in 88 isolates (86%). Seventy-two (71%) patients had eradicable foci, and foci were removed in most of them (92%, 66/72). The eradicable foci were not removed in the following cases: two cases of chemo-port, one case of Hickman catheter, one case of prosthetic valve, one case of vascular graft, and one case of pacemaker and vascular graft. The median interval from bacteremia to removal was 2 (IQR, 0–4) days (Table 2).

**TABLE 2:** Microbiological characteristics of heterogeneous vancomycin-intermediate *Staphylococcus aureus* isolates^*b*^

Characteristics	Total, *n* = 102	Vancomycin success, *n* = 22	Vancomycin failure, *n* = 80	*P-*value
Antimicrobial resistance				
Clindamycin	94 (92)	19 (86)	75 (94)	0.20
Ciprofloxacin	96 (94)	20 (91)	76 (95)	0.61
Erythromycin	96 (94)	20 (91)	76 (95)	0.61
Fusidic acid	79 (78)	15 (68)	64 (80)	0.13
Gentamicin	74 (73)	15 (68)	59 (74)	0.47
Rifampicin	16 (16)	4 (18)	12 (15)	0.74
Trimethoprim/sulfamethoxazole	11 (11)	5 (23)	6 (8)	0.06
Tetracycline	80 (78)	19 (86)	61 (76)	0.48
Sequence type				
ST5	85 (83)	16 (73)	69 (86)	0.19
ST72	7 (7)	2 (9)	5 (6)	0.64
ST239	8 (8)	4 (18)	4 (5)	0.06
*agr* genotype^*a*^				0.09
I	15 (15)	6 (27)	9 (12)	
II	85 (85)	16 (73)	69 (88)	
*agr* dysfunction	88 (86)	18 (82)	70 (88)	0.50
Vancomycin MIC				
Automated test				0.39
≤1.0 mg/L	52 (51)	13 (59)	39 (49)	
≥2.0 mg/L	50 (49)	9 (41)	41 (51)	
E-test				0.89
≤1.0 mg/L	15 (15)	3 (14)	12 (15)	
1.5 mg/L	46 (45)	10 (46)	36 (45)	
≥2.0 mg/L	41 (40)	9 (41)	32 (40)	
Broth microdilution method				0.07
≤1.0 mg/L	57 (56)	16 (73)	41 (51)	
1.5 mg/L	34 (33)	5 (23)	29 (36)	
≥2.0 mg/L	11 (11)	1 (5)	10 (13)	

^*a*^ Two isolates were non-typeable.

^*b*^ ST, sequence type; MIC, minimal inhibitory concentration.

### Comparison between the vancomycin failure and vancomycin success groups

Patients in the vancomycin treatment failure group were older than those in the success group [51 (IQR, 48–59) vs 68 (IQR, 59–73)], with statistical significance (*P* < 0.001). Sex and place of acquisition were comparable between the two groups. Hypertension [5 (23%) vs 42 (53%); *P* = 0.01], solid cancer [4 (18%) vs 34 (43%); *P* = 0.04], and ischemic heart disease [0 vs 16 (20%); *P* = 0.02] were more frequent in the vancomycin treatment failure group than in the vancomycin treatment success group. Liver cirrhosis [5 (23%) vs 5 (6%); *P* = 0.04] and solid organ transplantation (SOT) [8 (36%) vs 4 (5%); *P* < 0.001] were more frequent in the vancomycin treatment success group than in the vancomycin treatment failure group. Charlson comorbidity index was higher in the vancomycin treatment failure group [2 (IQR, 1–3) in the success group vs 3 (IQR, 2–5) in the failure group; *P* = 0.01], while McCabe Jackson classification was not different between two groups. History of a recent operation (within 1 month before bacteremia) was more frequent in the success group [15 (68%) vs 27 (34%) in the failure group; *P* = 0.004]. No differences in the presence of foreign materials, including CVC, catheters other than vascular catheters, and non-catheter foreign materials, were observed between the two groups. MRSA colonization was not significantly different between the two groups. The APACHE II scores at bacteremia were higher in the vancomycin failure group [11 (IQR, 9–15) in the success group vs 17 (IQR, 13–22) in the failure group; *P* < 0.001]. The Pitt bacteremia score was also higher in the vancomycin failure group [0 (IQR, 0–1) in the success group vs 1 (IQR, 0–3) in the failure group; *P* = 0.07]. The incidence of septic shock was comparable between the two groups. The sites of infections were not significantly different between the two groups. However, pneumonia and infective endocarditis were not observed in the vancomycin success group. The presence of eradicable foci and the removal rate of eradicable foci were comparable between the two groups (Table 1).

Vancomycin MIC using the BMD method was different between the two groups, isolates with vancomycin MIC ≤ 1.0 mg/L were more frequently observed in the vancomycin success group, and isolates with vancomycin MIC ≥ 2.0 mg/L were more frequently observed in the vancomycin failure group (*P* = 0.07). However, vancomycin MIC using E-test and automated test was not significantly different between the two groups. The resistance rate to antimicrobial agents was not significantly different between the two groups, except trimethoprim/sulfamethoxazole [5 (23%) in the success group vs 6 (8%) in the failure group; *P* = 0.06]. Most of the isolates were ST5, *agr* type II, and *agr* dysfunction in both groups. However, ST239 was more common in the vancomycin treatment success group [4 (18%) in the success group vs 4 (5%) in the failure group; *P* = 0.06]. There were no significant differences in *agr* genotype and *agr* functionality between the two groups (Table 2).

### Risk factors for vancomycin failure among patients with hVISA

Univariate logistic regression analyses conducted to determine the risk factors for treatment failure identified age [odds ratio (OR), 1.10; 95% confidence interval (CI), 1.05–1.16], hypertension (OR, 3.76; 95% CI, 1.26–11.17), solid cancer (OR, 3.33; 95% CI, 1.03–10.72), Charlson comorbidity index (OR, 1.53; 95% CI, 1.13–2.07), and APACHE II score at bacteremia (OR, 1.19; 95% CI, 1.07–1.32) to be associated with vancomycin failure. Removal of eradicable foci was not related to vancomycin treatment failure. Liver cirrhosis (OR, 0.23; 95% CI, 0.06–0.87), SOT (OR, 0.09; 95% CI, 0.02–0.35), and recent operation (OR, 0.24; 95% CI, 0.09–0.65) were associated with vancomycin success. We also included the variables with a *P*-value < 0.1, such as MRSA colonization, Pitt bacteremia score, vancomycin MIC ≤ 1.0 mg/L, ST239, and resistance to trimethoprim/sulfamethoxazole, in the multivariate logistic regression analysis. The multivariate analysis revealed that age [adjusted odds ratio (aOR), 1.15; 95% CI, 1.05–1.26] and APACHE II score at bacteremia (aOR, 1.16; 95% CI, 1.02–1.31) were risk factors for vancomycin treatment failure. SOT (aOR, 0.02; 95% CI, 0.001–0.34) and vancomycin MIC ≤ 1.0 mg/L (aOR, 0.17; 95% CI, 0.03–0.91) were associated with vancomycin treatment success (Table 3).

**TABLE 3:** Risk factors for vancomycin failure in the patients with heterogeneous vancomycin-intermediate *Staphylococcus aureus* bacteremia^*b*^

	Univariate analysis results [OR (95% CI)]	Multivariate analysis results^*a*^	
		Adjusted OR (95% CI)	*P-*value
Age	1.10 (1.05–1.16)	1.15 (1.05–1.26)	0.003
Hypertension	3.76 (1.26–11.17)		
Solid cancer	3.33 (1.03–10.72)	4.44 (0.89–22.04)	0.07
Liver cirrhosis	0.23 (0.06–0.87)		
Charlson’s comorbidity index	1.53 (1.13–2.07)		
MRSA colonization	2.37 (0.89–6.28)		
Recent surgery	0.24 (0.09–0.65)	0.20 (0.04–1.05)	0.06
Solid organ transplantation	0.09 (0.02–0.35)	0.02 (0.001–0.34)	0.01
Immunosuppressed status	0.42 (0.15–1.14)	11.73 (0.68–201.97)	0.09
APACHE II score at bacteremia	1.19 (1.07–1.32)	1.16 (1.02–1.31)	0.02
Pitt bacteremia score	1.41 (0.99–1.99)		
ST239 type	0.24 (0.05–1.04)		
Resistance to TMP/SMX	0.28 (0.08–1.01)		
Vancomycin MIC ≤ 1.0 mg/L	0.39 (0.14–1.11)	0.17 (0.03–0.91)	0.04

^*a*^ The model fitted the data well in terms of discrimination (*C*-statistic = 0.93) and calibration (Hosmer–Lemeshow goodness of fit statistic = 4.01, *P* = 0.86).

^*b*^ TMP/SMX, trimethoprim/sulfamethoxazole.

## DISCUSSION

The hVISA phenotype was detected in 19% of MRSA isolates, and 78% of these patients failed vancomycin treatment. CRBSI, primary bacteremia, and pneumonia were the most common sites of infection. Patients who failed with vancomycin treatment were older and had solid cancer and ischemic heart diseases more frequently than those who were successfully treated. Liver cirrhosis and SOT were more frequent in the vancomycin treatment success group. Charlson comorbidity index and APACHE II scores at bacteremia were higher in the vancomycin treatment failure group. Vancomycin MIC using the BMD method was higher in the vancomycin treatment failure group. Independent risk factors for vancomycin treatment failure were determined to be old age and severity of bacteremia. SOT and lower vancomycin MIC were associated with successful vancomycin treatment.

Previously reported mortality in hVISA infections ranged from 30% to 57% (10, 12, 14). Reported mortality was variable according to the infection focus and definition of mortality. In the present study, all-cause mortality was 68%, which was slightly higher than that in previous studies. The treatment failure rate was 78% in the present study, similar to previous studies that reported a vancomycin failure rate of 60%–82% in hVISA infections (3, 13). Because of the differences in study population, outcome definition, and microbiologic characteristics, there was a limitation in direct comparison between studies. However, there were no significant differences between the previous studies in clinical outcomes, including mortality and treatment failure rate.

Old age was an independent risk factor for vancomycin failure. In previous studies, old age was associated with poor clinical outcomes, such as treatment failure and mortality, in patients with MRSA infection (16, 17). The aging process is associated with diverse comorbidities and a decrease in immunity (18). Our result was in line with those of previous studies.

The severity of infection was also determined to be an independent risk factor for vancomycin treatment failure. The severity of infection was associated with poor outcomes in MRSA infections. Moreover, this finding was also observed in hVISA infections (8, 10, 14, 15). Our results support the results of the previous studies.

SOT was associated with vancomycin success in the present study. However, because the patients who underwent SOT should receive immunosuppressive therapy, SOT could be related to poor outcomes when we consider the relationship between immunosuppression and poor outcomes of *S. aureus* infection (17). When we compare the patients who underwent SOT and who did not, patients who underwent SOT were younger than the patients who did not [53 (IQR, 49–61) vs 67 (IQR, 57–72)]. Furthermore, sites of infection in SOT patients were CRBSI in seven patients, surgical site infection in two patients, and primary bacteremia in two patients. High-inoculum infections, such as bone and joint infections and infective endocarditis, were not observed in SOT patients. High-inoculum infections have been reported to be risk factors for treatment failure in MRSA infections (19, 20). Because appropriate adjustments were not made for several confounding factors, the relationship between SOT and vancomycin failure in hVISA bacteremia remains unclear.

Vancomycin MIC ≤ 1.0 mg/L was independently associated with vancomycin treatment success. Higher vancomycin MIC of MRSA isolates was related to poor clinical outcomes, including mortality and treatment failure, in a meta-analysis (21). Furthermore, a higher vancomycin MIC was also reported to be a risk factor for persistent bacteremia in patients with hVISA bacteremia (20). Vancomycin MIC seems to be an important risk factor for vancomycin failure not only in MRSA infection but also in hVISA infection. When we treat the MRSA infection with high vancomycin MIC, treatment failure should be closely monitored. However, because of limited studies on treatment options for MRSA infection with high vancomycin MIC, further studies on appropriate treatment in such cases are needed.

The research laboratory did not report the laboratory results to clinicians in the present study. Therefore, clinicians choose vancomycin as the definitive antibiotic based on the automated test. Using the automated test, vancomycin MIC of all isolates was equal to or lower than 2.0 mg/L, and all isolates were determined to be susceptible to vancomycin according to the Clinical Laboratory Standards Institute (CLSI) criteria. Because of the discordance between the automated test and the BMD method, vancomycin could be administered to patients with vancomycin non-susceptible isolates (22–24). However, the effect on the results could be limited because only one isolate was determined to have a vancomycin MIC of 4.0 mg/L using the BMD method, and the proportion of isolates with a vancomycin MIC ≤ 1.0 mg/L was similar between the automated test and the BMD method. Because most clinical laboratories do not use the BMD method, our results reflect real-world practice.

The automated test, E-test, and BMD method were used to identify vancomycin MIC. The BMD method is the gold standard for testing vancomycin MIC. However, most clinical laboratories use automated tests and E-test, owing to the labor-intensive nature of the BMD method (22). In previous studies, the E-test was more reliable than the automated tests for the results of the BMD method (22–24). However, the vancomycin MIC results of the E-test were higher than those of the BMD test, and the agreement between the E-test and the BMD test was suboptimal (23). In the present study, vancomycin MIC using the BMD method was an independent risk factor for vancomycin failure in patients with hVISA bacteremia. Despite the realistic limitations of the BMD method, using the BMD method in specific settings should be considered.

Isolates with ST239 were more frequently observed in the vancomycin treatment success group. In a previous study, Chong et al. (14) reported that the ST239 hVISA strain was associated with reduced mortality in patients with hVISA bacteremia. Because of the small proportion of ST239 strains in the present study, we could not evaluate the effect of ST on vancomycin failure. To evaluate the effect of ST on clinical outcomes, several studies are needed.

The present study has a few limitations. First, we included patients who received only vancomycin for over 7 days. Because the patients who died before completing 7 days of vancomycin treatment were not included in the study, the vancomycin failure rate could be underestimated, and a selection bias could have occurred. However, only two patients died within 7 days of vancomycin treatments among the patients who were excluded from using vancomycin for less than 7 days. Therefore, their effect on results could be limited. Second, the number of patients included in the study was small. Therefore, evaluation of the effect of ST and rare infection focus, such as infective endocarditis, on treatment failure could be limited. Despite these limitations, old age and the severity of infections showed a significant relationship with vancomycin failure. Moreover, lower vancomycin MIC was significantly related to vancomycin success.

In conclusion, the prevalence of hVISA among MRSA isolates was determined to be 19% and vancomycin treatment failed in 78% of hVISA bacteremia. Old age and severity of infection were independent risk factors of vancomycin treatment failure. SOT and low vancomycin MIC (≤1.0 mg/L) were related to vancomycin success. Vancomycin MIC determined using the BMD method is an important factor for vancomycin treatment failure, and its use should be considered in hVISA bacteremia.

## MATERIALS AND METHODS

### Study population

A prospective observational study was conducted from August 2008 to June 2020 at Asan Medical Center, a 2,700-bed tertiary-care hospital in Seoul, South Korea. Patients older than 18 years with *S. aureus* bacteremia were enrolled in the study. Methicillin-resistant isolates were tested for the hVISA phenotype. Patients with hVISA bacteremia who were treated with vancomycin over 7 days, as definitive therapy, were included in the present study. Patients with polymicrobial bacteremia and those discharged before positive blood culture results were excluded.

Vancomycin treatment failure was defined as the presence of either persistent bacteremia, recurrent bacteremia, or all-cause mortality. Persistent bacteremia was defined as bacteremia lasting more than 3 days despite vancomycin treatment, as longer durations have been linked to poor outcomes (25). Recurrent bacteremia was defined as a repeated bout of bacteremia by MRSA having identical antimicrobial susceptibility after negative blood culture and initial clinical improvement. Patients not meeting these criteria were considered successfully treated with vancomycin.

The study compared the clinical and microbiological characteristics of patients who were successfully treated with vancomycin (vancomycin success group) and those who were not (vancomycin failure group). Thereafter, the risk factors for vancomycin failure in patients with hVISA bacteremia were determined.

### Clinical data and definition

Baseline characteristics, clinical characteristics, and clinical outcomes were evaluated. The baseline characteristics included age, sex, place of acquisition, underlying diseases and conditions, severity of underlying diseases, and colonization of MRSA. Place of acquisition was classified as community-acquired, healthcare-associated, or hospital-acquired as described by Friedman et al. (26). An operation that was conducted within 1 month before bacteremia was defined as a recent operation. Neutropenia was defined as an absolute neutrophil count <500/µL. Immunosuppression status, with details of immunosuppressant use, steroid use, chemotherapy, and radiotherapy, was investigated. The severity of the underlying diseases was evaluated using the McCabe and Jackson classification and the Charlson comorbidity index (27). The presence of a CVC and other foreign materials, including catheters, such as urinary and biliary catheters, and non-catheters, such as prosthetic valves, prosthetic joints, and vascular grafts, at the time of bacteremia was determined. The site of infection was defined according to the Centers for Disease Control and Prevention criteria (28). Modified Duke’s criteria were used to determine infective endocarditis (29). Primary bacteremia was defined when the focus of infection was not observed, despite fastidious evaluation. The severity of bacteremia was evaluated using the Pitt bacteremia score, the presence of septic shock, and the APACHE II score (30, 31). We evaluated the presence of eradicable foci and the removal rate of eradicable foci. Eradicable foci were considered when patients had foreign materials at the infected site or abscess. Removal of eradicable foci was defined as the removal of foreign materials from the infected site or drainage of the abscess.

### Microbiological data

*S. aureus* isolates obtained from the initial blood cultures were analyzed for microbiological characteristics such as antimicrobial susceptibility, multilocus sequence typing (MLST), *agr* genotype, and *agr* functionality. Methicillin resistance was evaluated by detecting the *mecA* gene using PCR. A population analysis profiling test was conducted for all MRSA isolates to detect the hVISA phenotype (7). The isolates were defined as hVISA when the ratio of the area under the viable count-vancomycin curve (AUC) of the test isolate vs the AUC of the reference strain (Mu3; ATCC700698) was over 0.9. Antimicrobial susceptibility tests were conducted using a MicroScan system (Dade Behring, West Sacramento, CA, USA) and the standard criteria of the CLSI M100 31st edition. The MIC of vancomycin was evaluated using the BMD method and E-test in our research laboratory. The results of the research laboratory were not reported to clinicians. Therefore, clinicians used vancomycin based on the results of an automated antimicrobial susceptibility test using the MicroScan system in the clinical laboratory. MLST was performed as previously described (32). The *agr* genotype was determined using a PCR-based assay (33), and to determine *agr* functionality, δ-hemolysin activity was measured as previously described (34).

### Statistical analysis

Comparisons between the vancomycin failure and success groups were conducted using the chi-square and Fisher’s exact tests for categorical variables and the Student’s *t*-test and Mann–Whitney test for continuous variables. Risk factors for vancomycin failure were evaluated using logistic regression analysis. Variables that showed a *P*-value < 0.1 in univariate analysis were included in the multivariate analysis. A *P*-value < 0.05 was considered statistically significant. All statistical analyses were performed using SPSS 23.0 (SPSS Inc., Chicago, IL, USA).
