# Erratum for Yun et al., “Risk factors for vancomycin treatment failure in heterogeneous vancomycin-intermediate *Staphylococcus aureus* bacteremia

**DOI:** 10.1128/spectrum.03314-24

**Published:** 2025-02-24

**Authors:** Ji Hyun Yun, Euijin Chang, Seongman Bae, Jiwon Jung, Min Jae Kim, Yong Pil Chong, Sung-Han Kim, Sang-Ho Choi, Sang-Oh Lee, Yang Soo Kim

## ERRATUM

Volume 12, no. 8, e00333-24, 2024, https://doi/10.1128/spectrum.00333-24. Page 9, Acknowledgments, line 3: “HV22C1234” should read “HV22C0209.”

